# Sinapine thiocyanate exhibited anti-colorectal cancer effects by inhibiting KRT6A/S100A2 axis

**DOI:** 10.1080/15384047.2023.2249170

**Published:** 2023-08-30

**Authors:** Yan Yang, Zhirui Zeng, Lian Li, Shan Lei, Yingmin Wu, Tengxiang Chen, Jinjuan Zhang

**Affiliations:** a Transformation Engineering Research Center of Chronic Disease Diagnosis and Treatment, Guizhou Medical University, Guiyang, Guizhou, China; b Department of Physiology, School of Basic Medicine, Guizhou Medical University, Guiyang, Guizhou, China; c Internal medicine, The Third Affiliated Hospital of Guizhou Medical University, Guiyang, Guizhou, China; d Department of Ergology, School of Basic Medicine, Guizhou Medical University, Guiyang, Guizhou, China

**Keywords:** colorectal cancer, sinapine thiocyanate, KRT6A, proliferation, mobility

## Abstract

Sinapine thiocyanate (ST), an alkaloid existed extensively in seeds of cruciferous plants, exhibits a number of pharmacological effects, including anti-inflammatory and anti-malignancy properties. However, it is still unknown what effects and molecular mechanisms ST has on colorectal cancer (CRC). In the current study, it was indicated that ST inhibited proliferation, colony formation, and apoptosis *in vitro*, as well as arrested the G1 phase of CRC cells. There was a significant repressive effects of ST on invasion and migration of CRC cells *in vitro*. RNA-sequencing indicated that 750 differentially expressed genes existed in CRC cells after ST treatment, and enrichment analysis demonstrated that ST obviously decreased the activation of keratinization pathways. Among DEGs enriched in keratinization, keratin 6A (KRT6A) was decreased the most significant, as well as its target gene S100 calcium-binding protein A2 (S100A2). Low expression of KRT6A and S100A2 signatures indicated a favorable prognosis in CRC patients. Moreover, we found overexpression of KRT6A relieved the inhibitory effects of ST in CRC cells. Furthermore, ST inhibited the CRC cell proliferation *in vivo*, and reduced KRT6A and KI67 expression in xenograft tumor. Taken together, we demonstrated that ST exhibited anti-CRC properties by inhibiting KRT6A/S100A2 axis. It is possible that ST can be used as a treatment for CRC.

## Introduction

In the world, colorectal cancer (CRC) has the third-highest mortality rate of all malignant tumors in the digestive system.^1^ Patients diagnosed with the localized stage have a 90% 5-year survival rate, while it is estimated to be 10% at a distant or regional stage. Moreover, patients with CRC who are diagnosed when the disease is at an advanced stage are less likely to survive.^2,3^ Therefore, clinicians face a significant challenge in treating CRC due to its aggressive nature. The clinical treatment of CRC is mainly surgical treatment, while chemotherapy is a widely used adjuvant treatment for patients with middle-advanced CRC. The commonly used first-line chemotherapy drugs are 5-fluorouracil (5-FU) and platinum.^4^ However, most patients have severe adverse reactions and are prone to relapse after chemotherapy.^5^ Therefore, it is necessary to screen functional compound molecules as new drug precursors for development and utilization.

Keratin 6A (KRT6A) belongs to the keratin gene family and contributes to the activation of follicular keratinocytes following wounding in physiological conditions.^6^ Furthermore, previous studies indicated that KRT6A can activate the SRC pathway, and promote the development of intestinal villus.^7^ Calcium sensors and modulators, such as A2 (S100A2), are involved in calcium signaling within cells.^8^ S100A2 is also a composed protein of keratoconus corneal proteome, co-expressed with and regulated by KRT6A.^9^ Several cancer types have been found to have dysregulation of KRT6A or S100A2. A study by Che *et al*. showed that lung cancer with overexpression of KRT6A has a poorer prognosis.^10^ Chen *et al*. found that KRT6A promoted the metastasis of bladder cancer cells by inhibiting miR-31-5p.^11^ Targeting KRT6A siRNAs have been shown to reduce the activation of catenin cascades, thereby suppressing nasopharyngeal carcinoma cell proliferation and metastasis.^12^ Wang *et al*. found that KRT6A expression was elevated in colon adenocarcinoma, and related to tumor grade.^13^ Zhang *et al*. indicated that S100A2 was highly expressed in endometrial carcinoma, and predicted a poor prognosis.^14^ Li *et al*. exhibited that S100A2 had the potential to promote the glycolysis and proliferation of CRC.^15^ Huang *et al*. demonstrated that inhibition of S100A2 can suppress the epithelial-mesenchymal transition (EMT).^16^ This evidence indicated that inhibition of KRT6A or S100A2 can suppress malignant phenotypes of cancers, and they are attractive therapeutic targets for cancer.

Herbal medicine and their active ingredients were reported to provide significant benefits to cancer patients, especially in enhancing survivability, through exhibiting anti-inflammatory, anti-angiogenesis and increasing immunity.^17,18^ Among active compounds of cruciferous plants, sinapine thiocyanate (ST) exhibited effects on suppressing inflammation and increasing antioxidant activity.^19^ A previous study indicated the patients with spontaneous hypertension benefit from ST treatment by preventing vascular endothelial damage.^20^ In our previous study, ST inhibits pancreatic cancer cell proliferation and mobility by up-regulating DNA damage-induced alpha and growth arrest genes.^21^ However, limited knowledge of the effects and molecular mechanisms of ST were identified on CRC.

The current study aimed at identifying the effects of ST on CRC cells, as well as exploring the specific molecular mechanisms involved. It was found that ST inhibited cell proliferation and mobility in CRC mice, while these effects may be KRT6A/S100A2 axis dependent. ST may be a promising drug for treating CRC.

## Materials and methods

### Cell culture, drug, and transient transfection

Human CRC cell lines containing RKO, HCT-15, and HCT 116 were acquired by the American Type Culture Collection (USA). All CRC cells were cultured at 37°C with 5% CO_2_ in Dulbecco’s modified Eagle’s medium (DMEM; Thermo Fisher Scientific, USA) with 10% fetal bovine serum (FBS; Invitrogen, USA). ST (cat no. HY-N045) and mitomycin C (cat no. HY-13316) were acquired by MCE (Wuhan, China) and dissolved in diluted DMSO solvent (10%) to store at −20°C prior to use. The KRT6A coding domain sequence was subcloned into the pSUPER.puro (Sangon Biotech, Shanghai, China) plasmid to construct KRT6A-overexpression (KRT6A-oe) plasmid. CRC cells in 6-well plates upon reaching 60% confluence were transfected with the KRT6A-on plasmid or the empty vector (NC) using Lipofectamine 2000 (Thermo Fisher Scientific, USA). In the following 12 hours, the medium was removed and replaced with fresh culture medium with 10% FBS.

### Cell count kit-8 (CCK-8)

The 96-well plates were seeded with CRC cells (3 × 10^3^ cells/well) which were afterward treated with varying concentrations (0, 12.5, 25, 50 and 100 μM) of ST for 24 h and 48 h. Thereafter, in each well, a total of 10 μL CCK-8 reagent (Solarbio, Nanjing, China) was added for 2 h. The spectrophotometer at 450 nm wave length was conducted to determine the proliferative rates.

### Colony formation assay

CRC cells were seeded in per well of six-well plates with the treatment with varying concentrations (0, 12.5, 25 and 50 μM) of ST for 48 h. Then, the cells were digested, re-set in the six-well plates at a density of 500 cells per well. Cell colonies were fixed with 4% paraformaldehyde after 12 days of culture, and a 0.5% crystal violet solution (Solarbio, Nanjing, China) was used to stain the cell colony. The stereograms of cell colony were generated using a camera.

### Flow cytometry analysis

The cell cycle distribution of CRC cells was measured by DNA Content Quantitation Assay kit (Solarbio, Nanjing, China). CRC cells were cultured in DMEM medium without FBS to synchronize them with the G1 phase for 24 h. Following the removal of the FBS-free medium, the cells were treated with 0, 12.5, 25, and 50 μM of ST in DMEM medium contained 10% FBS for 48 h. CRC cells were soaked overnight in 70% ethanol at − 20°C environment in order to immobilization. Immobilized cells were washed twice with PBS and stained with propidium iodide element. For apoptosis assay, a 48-hour treatment of CRC cells with varying concentrations (0, 12.5, 25, and 50, M) of ST in DMEM with 10% FBS was conducted. Afterward, cells were collected and apoptosis rates were measured using the Annexin V-PE apoptosis Assay Kit (KeyGen BioTECH, Jiangsu, China). Flow Jo (version 7.6.1) was used for analysis of the flow cytometry results.

### Western blotting

Total protein in CRC cells were obtained via a supercentrifugal process using a radioimmunoassay precipitation lysis reagent (Solarbio, Nanjing, China) with 1% phenylmethylsulfonyl fluoride (Solarbio, Nanjing, China). Bicinchoninic acid quantification kit (Servicebio, Wuhan, China) was conducted to measure the concentration of samples. The proteins of different molecular weights in samples were separated on 12% SDS-polyacrylamide gels (Meilune, Dalian, China) for 1.5 h, and transferred into PVDF membranes (Thermo Scientific, USA). Skim milk powder (Beyotime Biotechnology, Suzhou, China) was applied to fill empty site in the membranes for 2 h. A 16-hour incubation at 4°C with anti-KRT6A (1:10000, Cat No. 10590–1-AP, Proteintech, Wuhan, China), anti-S100A2 (1:1000, Cat No. A12647, ABclonal, Wuhan, China), anti-N-cadherin (1:1000, Cat No. 22018–1-AP, Proteintech), anti-E-cadherin (1:1000, Cat No. 22018–1-AP, Proteintech), anti-P21 (1:2000, Cat No. 10355–1-AP, Proteintech), anti-cyclin B1 (1:2000, Cat No. 55004–1-AP, Proteintech), anti-caspase 7 (1:500, Cat No. 27155–1-AP, Proteintech), anti-cleave-caspase 7 (1:1000, Cat No. 8438, CST, USA), anti-caspase 8 (1:500, Cat No. A19549, Abconal, Wuhan, China), anti-cleave-caspase 8 (1:1000, Cat No. 8592, CST), anti-caspase 9 (1:500, Cat No. A2636, Abconal, Wuhan, China), anti-cleave-caspase 9 (1:1000, Cat No. 7237, CST) and anti-α-tubulin (1:1000, Cat No. AC039, ABclonal) was performed. Following twice wash with 0.1% Tween-20-containing Tris-buffered saline, chemiluminescence reagents (Solarbio, Nanjing, China) were configurated and utilized to visualize protein bands. The relative protein expression levels of KRT6A, S100A2, N-cadherin, E-cadherin, P21, cyclin B1 were referred to α-tubulin, while cleave-caspase 7, cleave-caspase 8 and cleave-caspase 9 were referred to caspase 7, caspase 8 and caspase 9, respectively.

### Wound healing assay

CRC cells (6 × 10^5^/per well) were cultured in six-well plates until 95% convergence degree. Wounds were constructed in monolayer cells using a pipette tip with a volume of 200-μL. Floating cells were removed after twice washing with PBS, and FBS-free medium contained varying concentrations (0 and 12.5 μM) of ST was added. In addition, mitomycin C (1 μM) was used to eliminate proliferative effects. Wound healing conditions were recorded for 0 to 24 h using an optical microscope (magnification 40×).

### Transwell assay

CRC cells were resuspended in FBS-free DMEM medium and adjusted the density as 1 × 10^5^ cells/ml. Then, 200 μL cell suspension was added into the upper transwell chamber (Corning, USA) pre-coated with Matrigel (Sigma-Aldrich, USA). The lower transwell chamber was also filled with 700 μL of DMEM medium containing 10% FBS. Varying concentrations (0, 12.5, 25, and 50 μM) of ST and mitomycin C (1 μM) was added for 24 h, a 30-minute immobilization and staining of invasive cells in the upper transwell chamber was then performed. PBS was used to remove residual crystal violet and cotton swabs were used to scrub noninvasive cells. To photograph each chamber’s invaded cells, an inverted microscope was used, while invasive cells in five fields were counted and equalized to assess the invasive ability of per group.

### RNA sequencing and bioinformatics analysis

Total RNA in cells which had been treated with diluent DMSO (0.05%) or ST (50 μM) for 24 h was extracted using TRIzol^TM^ Reagent (Sigma-Aldrich, USA). Bioanalyzer 2100 and RNA 6000 Nano LabChip kits(Agilent, CA, USA) were used to quantify RNA quantities and purity, and conducted to perform sequencing on high-quality RNA samples with RIN numbers > 7.0. Following the purification of the mRNA, mRNA fragments were reverse transcribed with SuperScript™ II Reverse Transcriptase (Invitrogen, USA).We sequenced a final cDNA library containing 300 ± 50 bp inserts according to the vendor’s protocol using an Illumina Novaseq™ 6000 (LC-Bio Technology CO., Ltd., Hangzhou, China). Count data profile of samples were analyzed using EdgeR package^22^ in R software after performing annotation of probe and batch normalization. Genes with LogFC ≥ 1 and P-value＜0.05 were selected as differentially expressed genes (DEGs). The changes of each genes were exhibited in a volcano plot. Up-regulated genes were exhibited as red dots, while down-regulated genes were set as blue dots. For performing biological function enrichment analysis, DEGs were exported into and analyzed in online database DAVID (https://david.ncifcrf.gov/). Terms with P < .05 were significant and visualized in a bubble diagram. Finally, change levels of genes were imported in Gene Set Enrichment Analysis (GSEA) software and genes were ranked according to the change levels. Gene order were matched in a presupposed reference, reactome term. *P* value < 0.05 was significant.

### Reverse transcriptase quantitative PCR

RNA was isolated from diluent DMSO (0.05%)-treated CRC cells and those treated with ST using TRIzol reagent. The PrimeScript 1st Strand cDNA Synthesis Kit (Takara, Japan) was used to synthesize complementary DNA (cDNA) from each sample using 2-μg total RNA. The SYBR Green Master Mix (Biosharp Life Sciences, Anhui, China) was used to detect gene expression by PCR in the Quant Studio5 fluorescence ration PCR instrument (Thermo Fisher Scientific, USA). A gene’s relative fold change was measured using the 2^−ΔΔCt^ method using α-tubulin as the reference gene. The primers used in the current study were presented as followed:

KRTAP2–3 forward primer: 5’-CTTTGCAGCCTAGCTGATCC-3’; KRTAP2–3 reverse primer: 5’-GGTGATGAGTCAGTGGGACA-3’; KRTAP2–1 forward primer: 5’-GGAACGCGTAACTCACCTTC-3’; KRTAP2–1 reverse primer: 5’-TTTTTGCAAGGCCAAAGAAC-3’; KRT38 forward primer: 5’-CTGTCTCTCCCATCGACATTGG-3’; KRT38 reverse primer: 5’-GTGTCCCACACTTGTCTGCC-3’; KRT5 forward primer: 5’- CCAAGGTTGATGCACTGATGG-3’; KRT5 reverse primer: 5’- TGTCAGAGACATGCGTCTGC-3’; KRT6A forward primer: 5’-CCAAGGCAGACACTCTCACA-3’; KRT6A reverse primer: 5’-GCAGCTCCTCGTACTTGGTC-3’; KRT34 forward primer: 5’-TCTGGTGGAAATTAACCGCAG-3’; KRT34 reverse primer: 5’-AGGAGTTGCCACTAGCATTGG-3’; KRT31 forward primer: 5’-TGTGGACCTGAATCGGGTG-3’; KRT31 reverse primer: 5’-CTGCGTGGTGAACCATTGC-3’; KRT13 forward primer: 5’-GACCGCCACCATTGAAAACAA-3’; KRT13 reverse primer: 5’-TCCAGGTCAGTCTTAGACAGAG-3’; α-tublin forward primer: 5’-ACCTTAACCGCCTTATTAGCCA-3’; α-tublin reverse primer: 5’-CACCACGGTACAACAGGCA-3’.

#### In vivo *assay*

*In vivo* assay was conducted in female BALB/c nude mice (age: 4 weeks) provided by Guizhou Medical University Animal Center. An injection of 200 μL of PBS containing 2 × 10^6^ HCT 116 cells was administered subcutaneously to each mice after 3 days of adaptive feeding in specified pathogen free environment. After 9 days, the mice then were randomly assigned to DMSO or ST treatment group (*n* = 5 for per group). A 40 mg/kg ST injection was given intraperitoneally per three days to the ST group, while control group was injected with isodose diluted-DMSO. During each three-day period, the tumor volume was calculated while the mice’s healthy status was monitored daily. All mice was euthanized at day 30, while tumor tissues were extracted for IHC detection. Animal Ethics Committee of Guizhou Medical University approved the animal experiment.

### Immunohistochemical staining

Following immobilization, dehydration, and embedding in paraffin, tumor tissue was sliced into 2-μm sections and stored. After deparaffinization with xylene and rehydration with alcohol, antigen retrieval was conducted by treatment with 1% sodium citrate reagent (Solarbio, Nanjing, China). Sections were incubated with H_2_O_2_ (Solarbio, Nanjing, China) and BSA (Solarbio, Nanjing, China) at room temperature for 2 h to avoid subsequent nonspecific binding. Washing PBS for twice, primary antibodies including anti-KRT6A antibody (1:400, Cat No. 10590–1-AP, Proteintech) and anti‐KI67 antibody (1:400; Cat No. 27309–1-AP, Proteintech) were utilized to incubated with tumor sections at 4°C for 16 h. Washing by PBS for twice, horse radish peroxidase conjugated secondary antibodies (Solarbio, Nanjing, China) was used to incubate with tumor sections at 20°C for 2 h. Diaminobenzidine (Solarbio, Nanjing, China) was used to recognize antibody-antigen complexes, while the immunological signals in tumor sections were obtained using an orthophoto microscope.

### Statistical analysis

The statistical analysis was conducted in SPSS 19.0 software. Using a Student’s t-test and a one-way analysis of variance with least significant difference t-test, differences between two and multiple groups were evaluated with the cutoff as *P* < .05.

## Results

### *ST suppressed CRC cell proliferation and colony formation* in vitro

**Figure 1.**
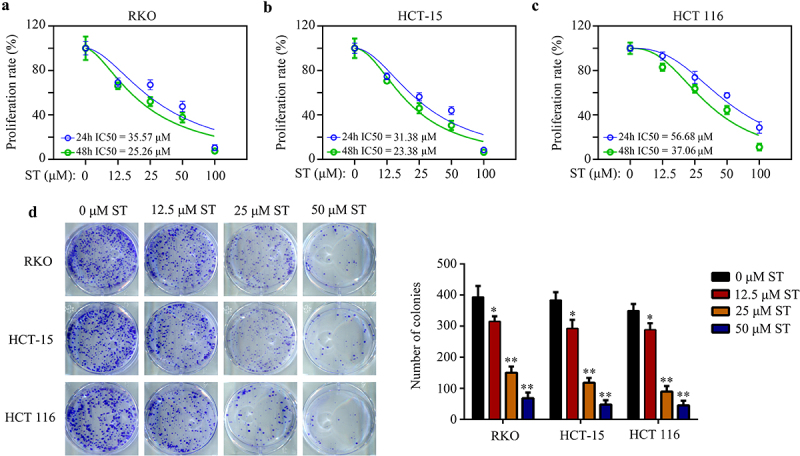
ST suppressed CRC cell proliferation and colony formation *in vitro*. (a – c) CCK-8 assays were used to detect the proliferative rates of RKO, HCT-115 and HCT 116 cells after ST treatment at 24 h and 48 h. (d) colony formation assays were used to the colony formation ability of RKO, HCT-115 and HCT 116 cells after ST treatment. *, *P* < .05; **, *P* < .01.

As determined by CCK-8 assays, ST reduced proliferation rates in RKO (24 h IC50 = 35.57 μM, 48 h IC50 = 25.26 μM), HCT-15 (24 h IC50 = 31.38 μM, 48 h IC50 = 23.38 μM), and HCT 116 cells (24 h IC50 = 56.68 μM, 48 h IC50 = 37.06 μM) (Figure 1a–c). Moreover, we found the ST treatment group formed fewer cell colonies than did the DMSO treatment group (Figure 1d).

### *ST induced the apoptosis of CRC cells* in vitro

**Figure 2.**
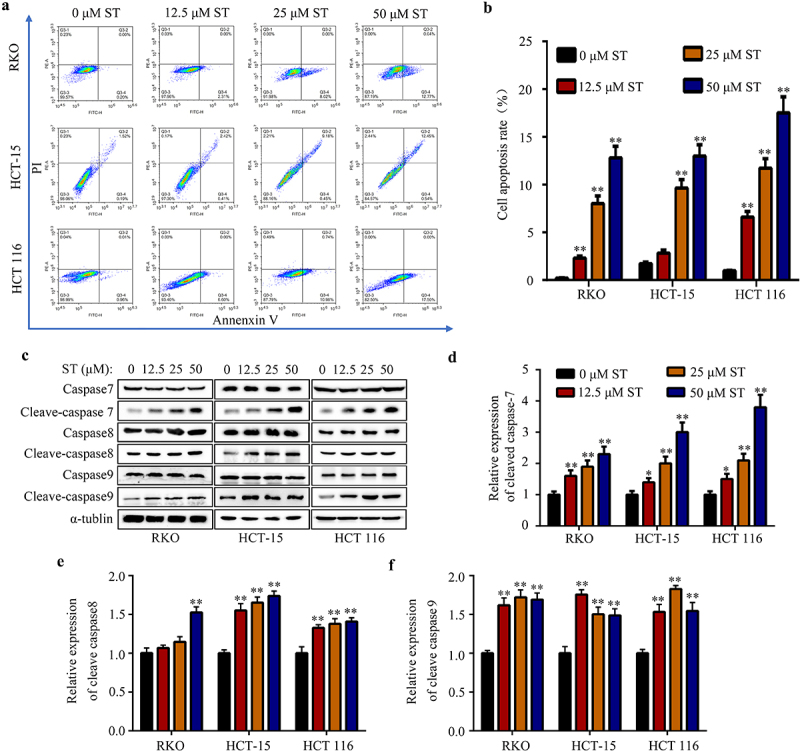
ST induced the apoptosis of CRC cells *in vitro*. (a-b) Flow cytometry was used to detect the apoptosis rate of RKO, HCT-115 and HCT 116 cells after ST treatment. (c-f) Western blotting was used to detect the expression of caspase 7 and cleave-caspase 7, cleave-caspase 8 and cleave-caspase 9 in RKO, HCT-115 and HCT 116 cells after ST treatment. *, *P* < .05; **, *P* < .01.

We then determined the apoptosis rate of CRC cells after ST treatment. Results indicated that ST significantly elevated the apoptosis rate of RKO, HCT-15, and HCT 116 cells *in vitro* (Figure 2a–b). Similarly, expression of the apoptosis biomarker cleave-caspase 7, cleave-caspase 8 and cleave-caspase 9 in CRC cells after treatment with ST were significantly increased (Figure 2c–f). These results indicated that ST has potential to induce the apoptosis of CRC cells *in vitro*.

### *ST induced the G1 phase arrest of CRC cells* in vitro

**Figure 3.**
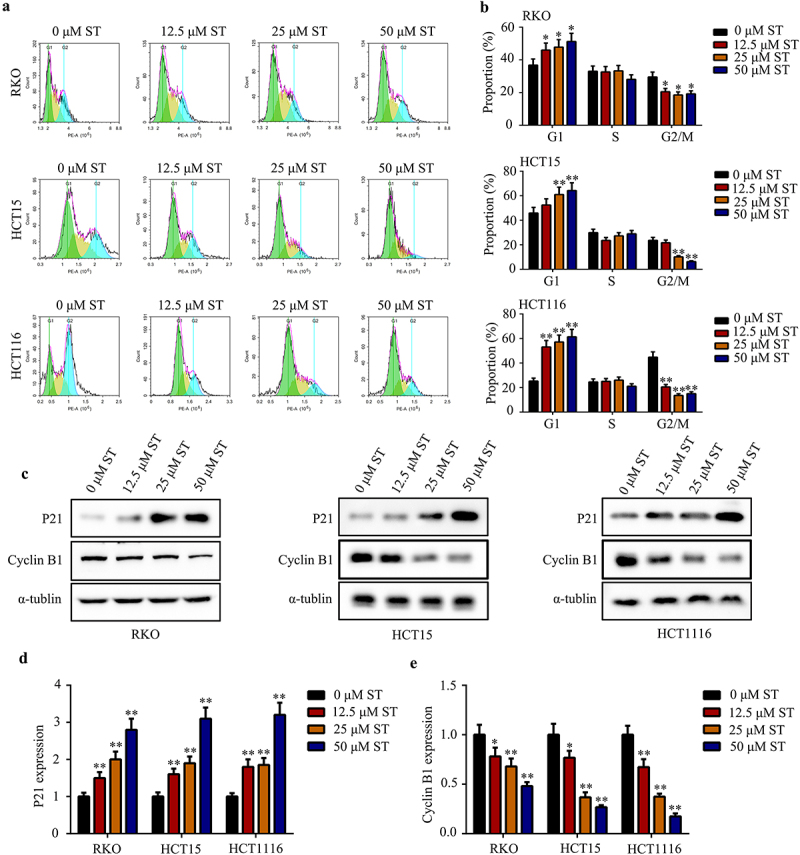
ST induced the G1 phase arrest of CRC cells *in vitro*. (a-b) Flow cytometry was used to detect the cell cycle of RKO, HCT-115 and HCT 116 cells after ST treatment. (c-e) Western blotting was used to detect the expression of P21 and cyclin B1 in RKO, HCT-115 and HCT 116 cells after ST treatment. *, *P* < .05; **, *P* < .01.

Cell cycle analysis indicated that, following ST treatment, cell proportion of CRC cells in G1 phase increased significantly, while those in G2/M phase decreased significantly (Figure 3a–b). Expression of the G1 phase checkpoint P21 and cyclin B1 was then checked in CRC cells, and results indicated that ST treatment elevated P21 expression in RKO, HCT-15, and HCT 116 cells while decreasing cyclin B1 expression (Figure 3c–d).

### *ST suppressed the mobility of CRC cells* in vitro

**Figure 4.**
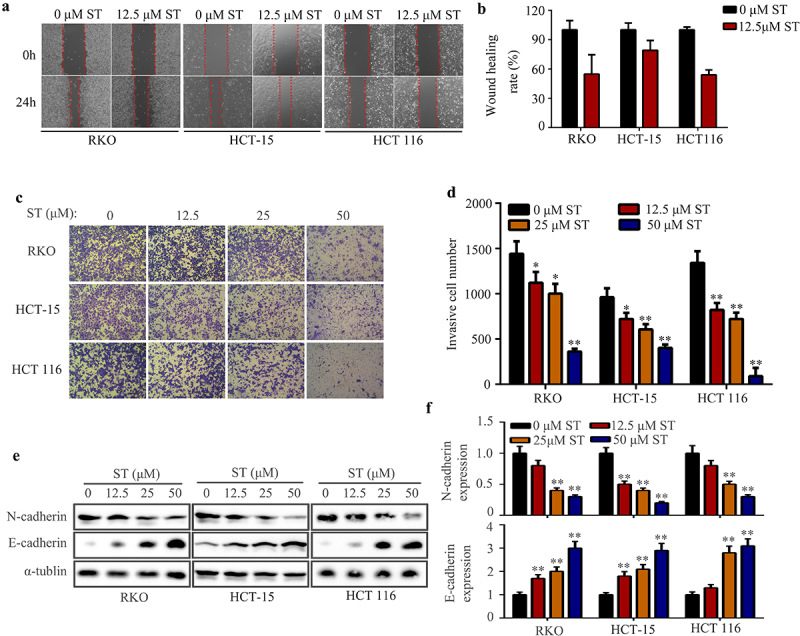
ST suppressed the mobility of CRC cells *in vitro*. (a-b) Wound healing assay was used to detect the migration rate of RKO, HCT-115 and HCT 116 cells after ST treatment. (c-d) transwell assay was used to detect the invasion rate of RKO, HCT-115 and HCT 116 cells after ST treatment. (e-f) Western blotting was used to detect the expression of N-cadherin and E-cadherin in RKO, HCT-115 and HCT 116 cells after ST treatment. *, *P* < .05; **, *P* < .01.

Based on wound healing assays, ST significantly inhibited the migration of RKO, HCT-15, and HCT 116 cells (Figures 4a–b). ST treated RKO, HCT-15 and HCT 116 cells also reduced their invasiveness in matrigel based on the transwell assay (Figure 4c–d). Previous studies demonstrated that EMT is a key process during cell metastasis. The expression of EMT biomarkers, E-cadherin and N-cadherin was subsequently measured after ST treatment. A significant reduction in N-cadherin expression and an increase in E-cadherin expression was observed in CRC cells treated with ST (Figure 4e–f).

### KRT6A was identified as a key target of ST in CRC cells

**Figure 5.**
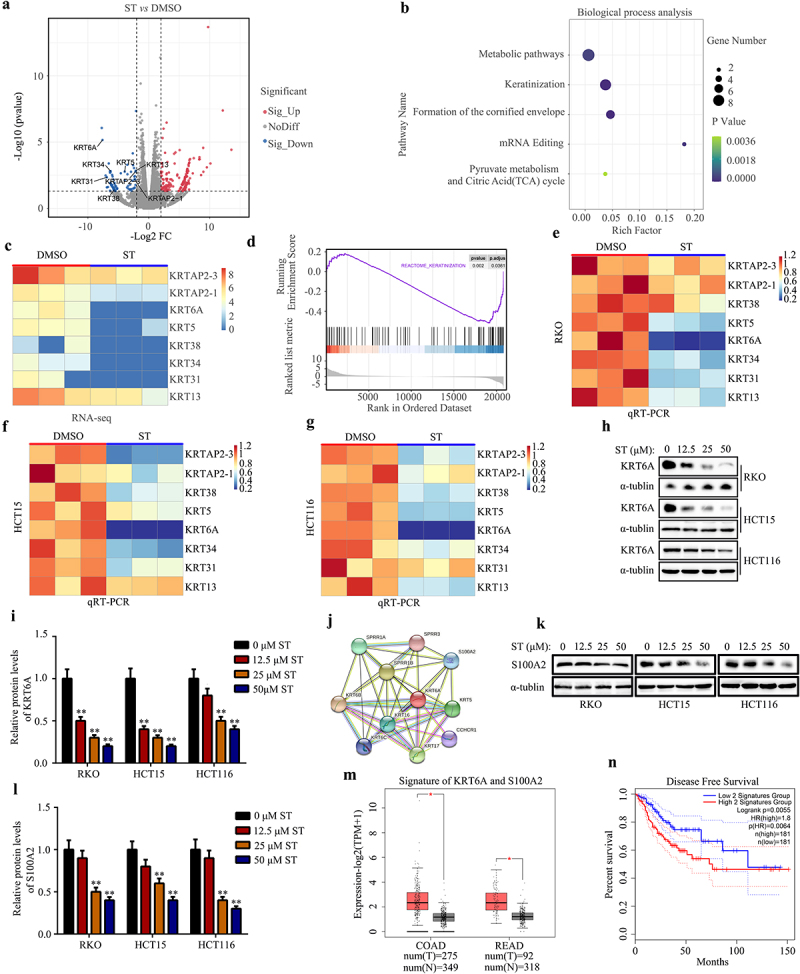
KRT6A was identified as a key target of ST in CRC cells. (a) DEGs between ST treatment group and DMSO treatment group were identified by RNA-sequencing. (b) biological process enrichment analysis for the DEGs. (c) Heatmap showed the decreased expression of members in keratinization after ST treatment based on RNA sequencing. (d) GSEA analysis was performed to determine the effect of ST on keratinization pathway. (e-g) the mRNA levels of members in keratinization after ST treatment in RKO, HCT-115, and HCT 116 cells. (h-i) protein levels of KRT6A in RKO, HCT-115, and HCT 116 cells after ST treatment. (j) Interacted proteins of KRT6A was exhibited. (k-l) protein levels of S100A2 in RKO, HCT-115, and HCT 116 cells after ST treatment. (m) signature of KRT6A and S100A2 in colonic adenocarcinoma (COAD) tissues, rectum adenocarcinoma (READ) tissues and corresponding non-tumor tissues. (n) the overall survival rate of patients with high and low signature of KRT6A and S100A2. *, *P* < .05; **, *P* < .01.

The DEGs between CRC cells treated with DMSO and ST (50 μM) was analyzed by RNA-sequencing to explore the mechanisms of ST in CRC cells. After analysis, total 750 DEGs in HCT 116 cells treated with ST were identified (*vs* DMSO; Figure 5a). Then, DAVID database was utilized to perform biological process (BP) enrichment analysis, we found that DEGs induced by ST were significantly enriched in the BP terms of “metabolic pathway”, “keratinization”, “formation of the cornified envelope”, “mRNA editing” and “pyruvate metabolism and citric acid cycle” (Figure 5b). Previous studies indicated that various members involved in keratinization play as oncogenes in CRC, therefore, we focused on them more. Among 750 DEGs, total 8 DEGs including KRTAP2–3, KRTAP2–1, KRT6A, KRT5, KRT38, KRT34, KRT31 and KRT13 were enriched in “keratinization” term. Interesting, we found that all of them were all decreased after ST treatment based on the RNA-sequencing results (Figure 5c). Further, we performed the GSEA analysis and found that ST had the potential to inhibit keratinization (Figure 5d).

We performed qRT-CRCR to measure the expression of members involved in keratinization after treatment of ST. Consistent with RNA-sequencing, we found that most of the members involved in the keratinization were most decreased. Among them, we found that KRT6A was reduced the most significantly in RKO, HCT-15 and HCT 116 cells (Figure 5e–g). Therefore, we consider that KRT6A may be a key target of ST in CRC cells. After ST treatment with different concentrations (12.5, 25 and 50 μM), western blot analysis was performed to determine protein expression of KRT6A in RKO, HCT-15, and HCT 116 cells. In these CRC cells, ST significantly reduced the protein expression of KRT6A (Figure 5h–i).We then used the STRING database to explore the downstream genes of KRT6A (Figure 5j). Among them, previous studies indicated that S100A2 is an oncogene in CRC.^23^ Therefore, we then determined whether the expression of S100A2 was also changed after ST treatment. Western blot analysis demonstrated that ST significantly reduced the expression of S100A2 in RKO, HCT-15, and HCT 116 cells (Figure 5k–l). Interesting, after calculating the comprehensive signature of KRT6A and S100A2 in CRC tissues (including colon adenocarcinoma and rectum adenocarcinoma), we found that the signature score derived from KRT6A and S100A2 was higher in CRC tissues compared non-tumor tissues (Figure 5m). Moreover, patients with higher signature scores had a shorter overall survival rate than those with lower signature scores. In conclusion, this evidence indicated that KRT6A and its downstream S100A2 may be key targets of ST.

### Suppressive effects of ST can be reversed by KRT6A- overexpression in CRC cells

**Figure 6.**
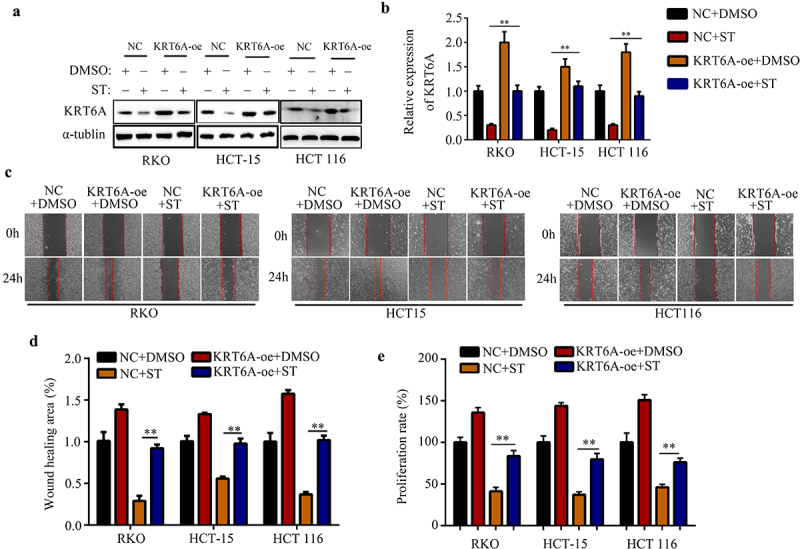
Suppressive effects of ST can be reversed by KRT6A- overexpression in CRC cells. CRC cells including RKO, HCT-115 and HCT 116 were treated as followed: negative control vector (NC) + DMSO, NC + ST, KRT6A-overexpression plasmid (KRT6A-oe) + DMSO and KRT6A-oe + ST. (a-b) expression of KRT6A in each group cells were determined by western blotting. (c-d) Wound healing assays were used to detect the migration rates in each group cells. (e) CCK-8 assays were used to detect the proliferative rates in each group cells. **, P < 0.01.

For determining whether KRT6A participates in ST functions in CRC cells, RKO, HCT-15 and HCT 116 cells were transfected with KRT6A-overexpression (KRT6A-oe) and empty vector (NC), and treated them with ST and isodose DMSO (Figure 6a–b). Cells after treatment were digested and re-set in six plate to perform the wound healing assays. Results revealed that RKO, HCT-15, and HCT 116 cells with KRT6A-oe exhibited stronger migration ability after ST treatment compared with NC cells (Figure 6c–d). Furthermore, CCK-8 results indicated that RKO, HCT-15, and HCT 116 cells with KRT6A-oe exhibited higher proliferation after ST treatment compared with those in NC groups (Figure 6e). Based on these results, KRT6A contributed to ST-induced biological functions.

### *ST reduced the proliferation of HCT 116* in vivo, *as well as reducing KRT6A expression in tumor tissues*

**Figure 7.**
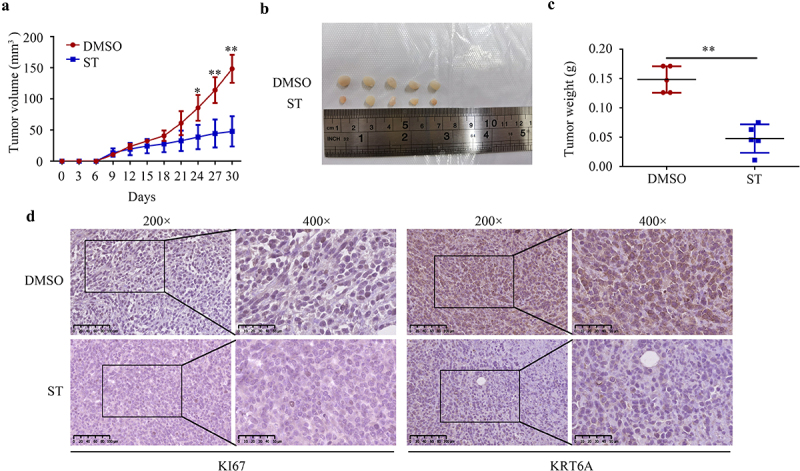
ST reduced the proliferation of HCT 116 in vivo, as well as reducing KRT6A expression in tumor tissues. (a-b) tumor volume of the tissues derived from HCT116 cells after ST or DMSO treatment. (c) Weight of the tissues derived from HCT116 cells after ST or DMSO treatment. (d) expression of KRT6A and KI67 in the tissues derived from HCT116 cells after ST or DMSO treatment. **, *P* < .01.

In order to examine the effects of ST on the proliferation of CRC cells *in vivo*, we subcutaneously injected HCT 116 cells into mice. *In vivo*, ST treatment significantly decreased CRC cell proliferation (Figure 7a–b) and tumor weight (Figure 7c) in comparison to DMSO treatment. Moreover, ST treatment remarkably reduced KI67 and KRT6A expression in tumor tissues compared with DMSO treatment (Figure 7d–e).

## Discussion

A poorly surviving malignancy, one with decreased quality of life in patients, diagnostic and therapeutic dilemma, CRC has an extremely detrimental impact on patients. Chemotherapy and surgery are the most common therapeutic strategies used for CRC.^24,25^ Due to severe side effects and unstoppable recurrences, these therapies are not effective for most patients with CRC. Developing new therapeutic methods and understanding the mechanisms of CRC progression are vital to its treatment.

Natural products have been shown to be effective at anti-tumor and to be low in toxicity. In recent years, the FDA has approved a growing number of molecules derived from medicinal plants that are bioactive as anticancer drugs.^26,27^ For example, omacetaxine mepesuccinate, an alkaloid isolated from *Torreya Grandis*, exhibited its anti-CRC effects via inhibiting EPH receptor B4.^28^ Berberine is an alkaloid isolated from the Chinese herbal medicine Huanglian, which exhibited its inhibitory effects on the proliferation of CRC cells at concentrations of 1.25 μM-160 μM.^29,30^ In the present study, as a quaternary amine alkaloid found in cruciferous plants, ST was found to has significant inhibitory effects on the proliferation, colony formation and mobility of CRC cells *in vitro*, as well as inducing G1 phase arrest and cell apoptosis. These were the first pieces of evidence indicating that ST had remarkable anti-CRC effects.

RNA-sequencing is an important tool for discovering the internal development mechanism of the tumor.^31^ Similarly, the mechanism of drug action can be evaluated comprehensively and objectively by RNA-sequencing followed by differentially expression gene analysis and enrichment analysis of the cells before and after drug treatment.^32^ To explore the molecular mechanism of ST, RNA-sequencing and relative experiments were performed, and the results indicated ST had the potential to inhibit the keratinization pathway, especially for the member KRT6A. Previous studies indicated that KRT6A and its downstream gene S100A2 were the biomarkers and therapeutic targets for various cancers, including CRC. Therefore, we determined whether KRT6A is involved in the molecular mechanisms of ST. Consistent with the RNA-sequencing, a significant reduction in KRT6A mRNA and protein levels was observed after ST treatment, as well as a decrease in the expression of S100A2 protein, downstream of KRT6A. Cells related to CRC were repressed by ST when KRT6A was overexpressed. As well as reducing KRT6A expression in tumor tissues, a significant suppressive effect of ST on CRC cell proliferation was observed *in vivo*. In conclusion, ST repressed CRC cell proliferation and mobility by inhibiting KRT6A/S100A2 axis. ST may be effective for CRC treatment.

## Abbreviations

CCK-8: cell count kit-8
EdU: 5-Ethynyl-2’- deoxyuridine
FBS: fetal bovine serum
KRT6A: keratin 6A
PBS: phosphate buffer saline
CRC: colorectal cancer
RT-qPCR: real-time quantitative polymerase chain reaction
ST: Sinapine thiocyanate

## Data validity

The corresponding author can be contacted for original experimental data when needed.
